# A Case of IgG4-related Sclerosing Mesenteritis

**DOI:** 10.7759/cureus.2147

**Published:** 2018-02-03

**Authors:** Zeeshan Butt, Syed H Alam, Oleksandr Semeniuk, Sonum Singh, Gurdeep S Chhabra, Irene J Tan

**Affiliations:** 1 Internal Medicine, Baystate Medical Center; 2 Department of Rheumatology, Temple University Hospital; 3 Internal Medicine, University of Maryland Prince George's Hospital Center; 4 Section of Rheumatology, Temple University Hospital

**Keywords:** sclerosing mesenteritis, igg4-related disease, abdominal mass, mesenteric mass

## Abstract

A 60-year-old African-American male presented to the emergency department with abdominal pain and distention associated with decreased appetite and weight loss for several weeks. A computed tomography (CT) scan of the abdomen and pelvis showed an 8 cm mesenteric mass with surrounding stranding and poorly defined borders. The patient underwent exploratory laparotomy and complete resection of the mass since the frozen section could not give a definite diagnosis. Histopathology showed fibro-adipose tissue with lymphoid hyperplasia, vague nodular collections of foamy histiocytes with giant cell reaction, marked chronic inflammation, fat necrosis, and prominent sclerosis/fibrosis. Methenamine silver and acid-fast stains were negative for fungal and mycobacterial organisms respectively. Examination of tissue with immunohistostains showed increased immunoglobulin G4 (IgG4)-positive plasma cells. Other features observed were scattered areas of phlebitis, pockets of tissue eosinophilia, and focal storiform fibrosis leading to the diagnosis of IgG4-related sclerosing mesenteritis. The patient did not require steroids after the surgical resection and was disease free at six-month follow up.

## Introduction

Immunoglobulin G4 (IgG4)-related sclerosing mesenteritis is one of the manifestations of a rather uncommon IgG4-related disease (IgG4-RD). It is characterized by chronic inflammation and fibrosis resulting from deposition of IgG4-positive plasma cells in affected tissues. Other organs commonly involved in IgG4-RD are pancreas, orbit, salivary glands, and retroperitoneal structures. Here, we present a case of IgG4-related sclerosing mesenteritis presenting with a mesenteric mass.

## Case presentation

A 60-year-old African-American male with a past medical history of a stab wound to the abdomen, post laparotomy years ago, presented to the emergency department with abdominal pain and distention for seven weeks. The pain was characterized as continuous, involving all quadrants, nagging, nonradiating, 7/10 in intensity, associated with decreased appetite, bloating, constipation, and weight loss of 50 lbs over seven weeks. The patient denied fever, chills, night sweats, nausea, vomiting, and blood in the stool. The patient endorsed shortness of breath on exertion and disturbed sleep due to abdominal distention. The patient had colonoscopy 1.5 years ago and reported it as normal. The patient denied tobacco, alcohol, illicit drug abuse, and he worked as a security guard. The patient’s vital signs were within normal limits and his physical examination was remarkable for abdominal distention with mild tenderness on deep palpation in all quadrants with rebound tenderness. Laboratory findings showed mild anemia with hemoglobin of 11.8 g/dL, hematocrit of 37%.

**Figure 1:**
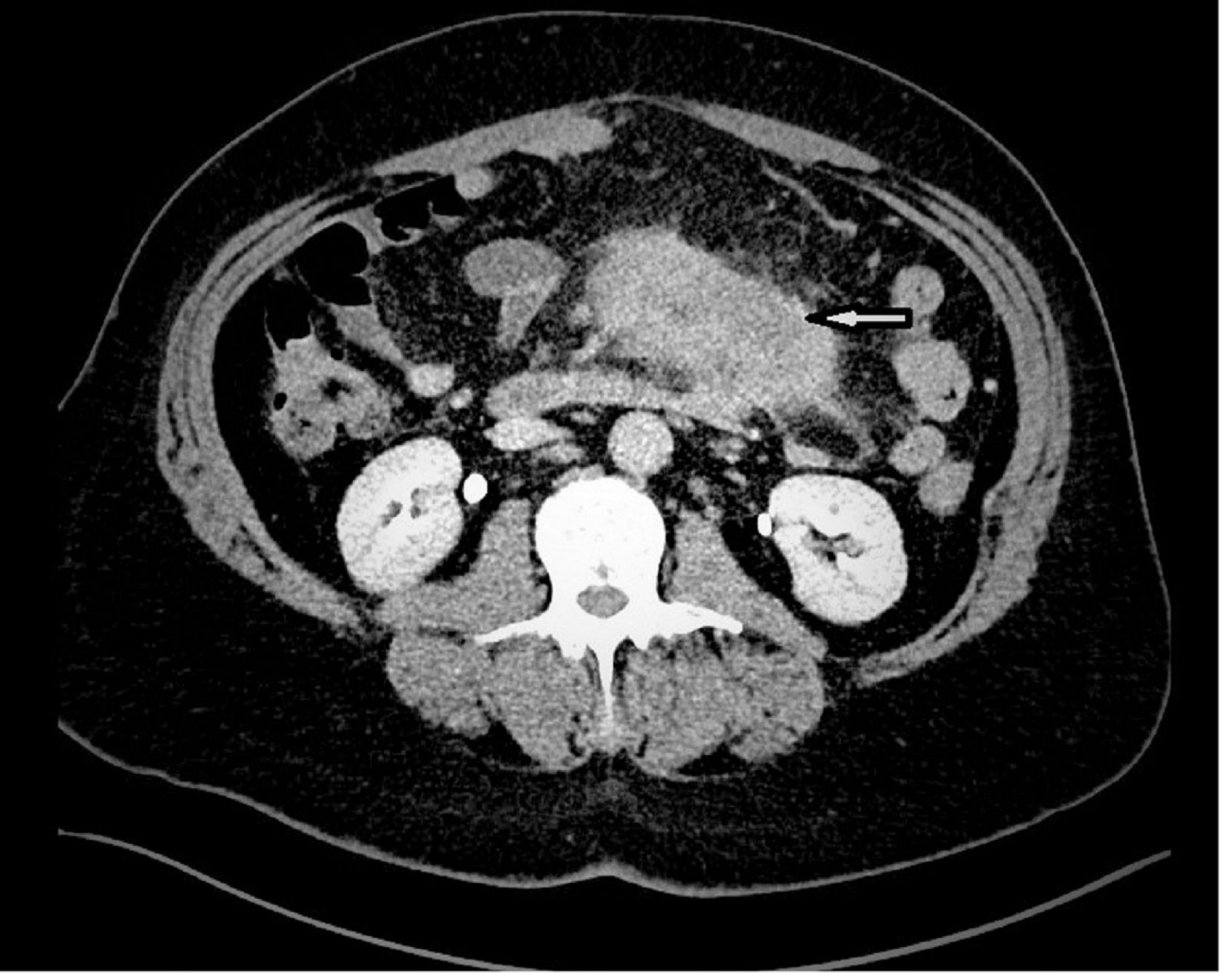
Computed tomography of the abdomen with intravenous contrast showing a large 8 cm mesenteric mass (white arrow) with surrounding stranding and poorly defined borders

The patient first underwent computed tomography (CT) scan of the abdomen and pelvis with intravenous contrast that showed an 8 cm mesenteric mass with surrounding stranding and poorly defined borders, no bowel ileus or obstruction but thickening of the descending colon through the sigmoid, with mild surrounding stranding (Figure 1).

**Figure 2:**
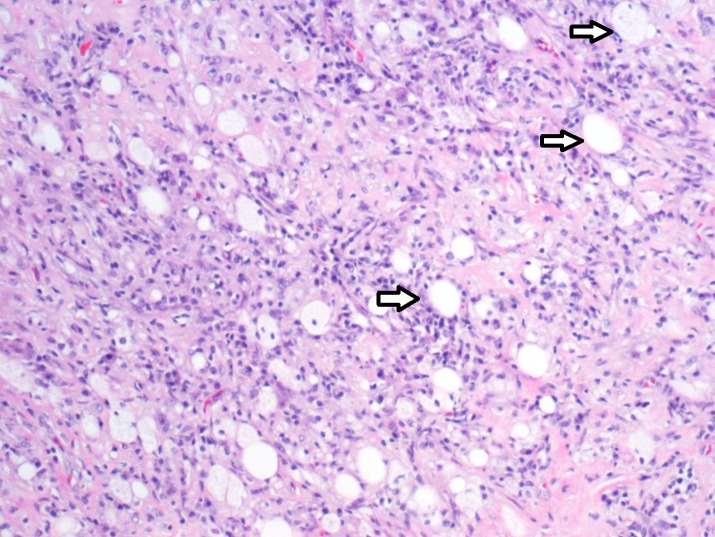
The tissue specimen from the mesenteric mass shows foamy histiocytes with fat necrosis (Haemotoxylin and Eosin staining)

Oncology and general surgery services were consulted and the decision was made to proceed with exploration laparotomy and biopsy of mesenteric mass with frozen section. No metastatic disease or ascites were noted. There was a mass at the base of the mesentery involving the transverse mesocolon overlying the middle colic as well as the superior mesenteric artery. The mass area was ligated and wedge resection was performed and sent for frozen section. Tru-Cut biopsies of the retroperitoneal area were also sent. The pathologist was unsuccessful in identifying a diagnosis intraoperatively as what was mostly seen was fibrosis. More specimen was required, hence careful dissection was started. The base of the mesentery was opened and the mass was then carefully dissected. Resection was continued along the mesentery to the small bowel where the mass, as well as the small bowel, was excised. The results of the preliminary biopsy of the mesenteric mass and omentum showed fibro-adipose tissue with lymphoid hyperplasia, vague nodular collections of foamy histiocytes with giant cell reaction, marked chronic inflammation, fat necrosis, and prominent sclerosis/fibrosis (Figure 2).

**Figure 3:**
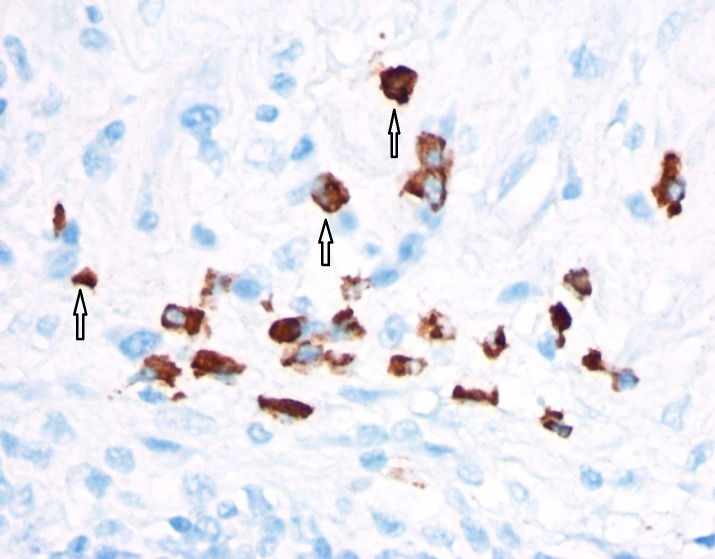
Immunostain of IgG4-positive plasma cells

Immunohistochemistry showed CD20 and paired box containing (PAX) 5-stained lymphoid follicles and scattered B cells, CD3-stained interfollicular areas and scattered T cells, and CD56 stained histiocytes (foam cells). Pan-cytokeratin stain was negative. Methenamine silver and acid-fast stains were negative for fungal and mycobacterial organisms respectively. Flow cytometry showed a mixed population of polytypic B-cells (32%) and T lymphocytes (49%) with no pan-T cells, no pan-T cell antigen deletion or B cell light chain restriction detected. Consultation with John Hopkins reference laboratory was obtained and immunohistostains done there showed increased IgG4-positive plasma cells (focally over 40 in one high power field) (Figure 3).

**Figure 4:**
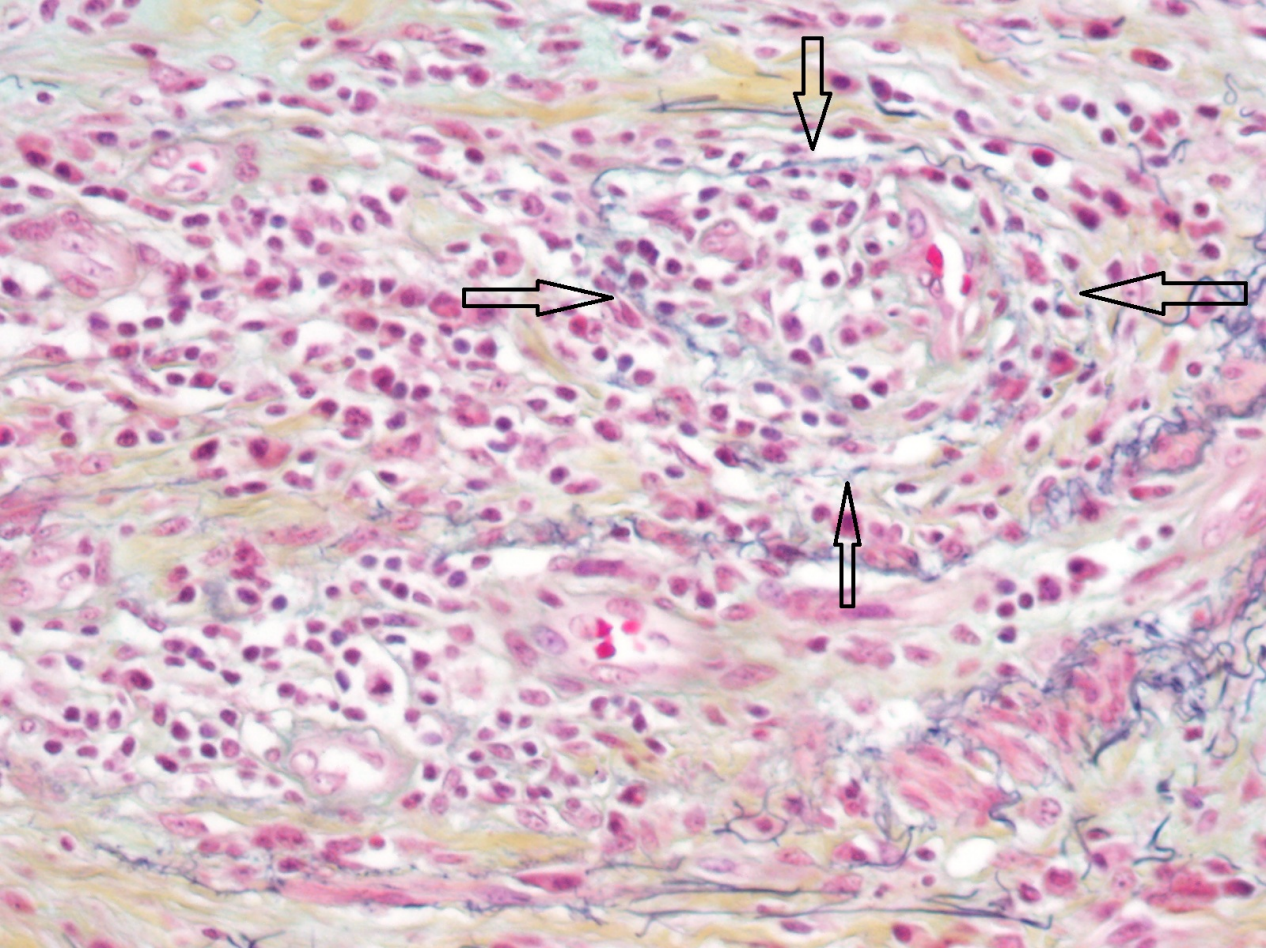
Obliterative phlebitis with the elastic fibers stained black (Movat stain)

A Movat stain highlighted areas of phlebitis (Figure 4).

**Figure 5:**
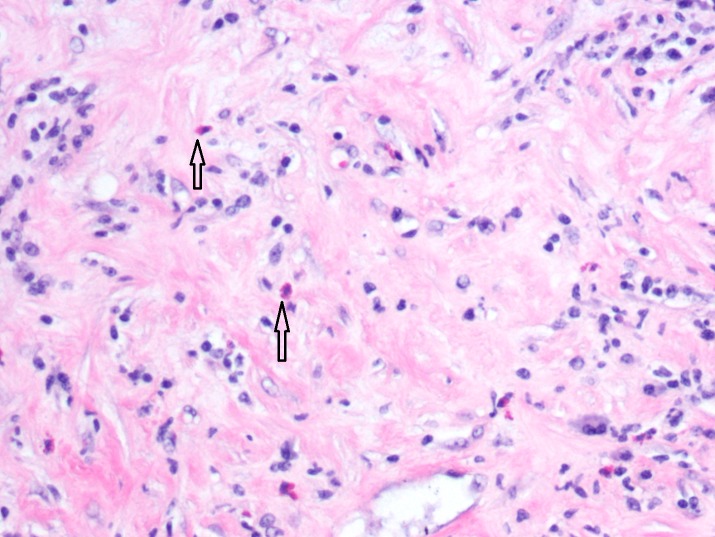
Scattered eosinophils (Hematoxylin and Eosin staining)

**Figure 6:**
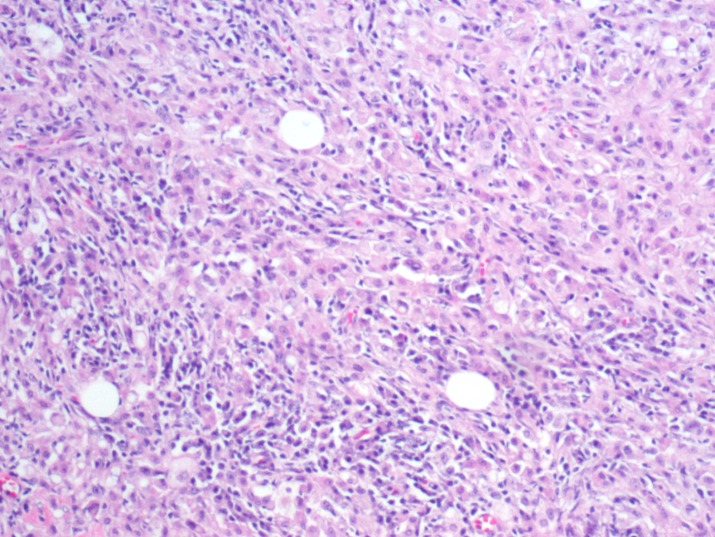
Infiltration of plasma cells and lymphocytes with storiform fibrosis (Hematoxylin and Eosin staining)

In addition, there were pockets of tissue eosinophilia and focal storiform fibrosis (Figures 5-6).

Even though there were focal areas of fat necrosis, the overall features argued in favor of IgG4-related disease. Plasma IgG4 level was measured a few days after the resection of the mass and it was normal at 47 mg/dL (reference range 4.0-86.0 mg/dL). The patient had a protracted hospital course requiring two additional laparotomies for resection of the ischemic small bowel.

The patient was discharged with oncology outpatient follow up. A CT scan of the abdomen with contrast at three months showed a residual soft tissue mass-like density within the mesenteric fat anterior to the third portion of the duodenum measuring 6.4 x 2.7 cm. A CT scan of the abdomen repeated six months after the surgery showed that the mass-like density in the mid-abdomen had mostly resolved.

## Discussion

IgG4-RD represents a relatively recently recognised disease that has several features: a tendency to form tumefactive lesions in multiple sites, a characteristic histopathological appearance, and often, but not always, elevated serum IgG4 concentrations [1]. The diagnosis of IgG4-RD rests on the combined presence of the characteristic histopathological appearance and increased number of IgG4+ plasma cells. The histopathological features critical for diagnosis are a dense lymphoplasmacytic infiltrate, a storiform pattern of fibrosis, and obliterative phlebitis [1].

Our patient represents a case of IgG4-related sclerosing mesenteritis. Pathologic descriptions and case reports of this clinicopathological entity started to appear in the literature in the last decade [2-8] but still remain anecdotal.

Clinically, patients with IgG4-related sclerosing mesenteritis present with similar symptoms as patients with classic sclerosing mesenteritis; chronic abdominal pain [3-4, 6-8] and related problems such as abdominal distention or discrete abdominal mass [2, 4, 8], nausea, vomiting, diarrhea, fever, anorexia, and weight loss [7]. Our patient had abdominal pain, abdominal distention, constipation, anorexia, and weight loss.

Usually, these patients are found to have an abdominal mass during the imaging of the abdomen and subsequently undergo laparotomy as diagnosis which is difficult to establish without pathological examination [1-4, 6-8].

The histologic characteristics of this mass included lymphoid hyperplasia in the mass, the presence of storiform fibrosis, and areas of phlebitis with vessel obliteration. There were, however, focal areas of fat necrosis but overall the tumor meets all three histopathological features for IgG4-RD [1]. There was also a marked increase in IgG4-positive plasma cells (focally over 40 in one high power field) in the tissue as well. Serum IgG4 level was not elevated in our case; however, it does not exclude the diagnosis of IgG4-RD [1, 9].

The treatment of IgG4-related sclerosing mesenteritis is not well established because of the rarity of the condition. However, there is an increasing data from case reports and case series on the treatment of IgG4-related autoimmune pancreatitis which is the most commonly diagnosed form of IgG4-RD, and also, we have historical data from case reports and case series of patients with “nonspecific” sclerosing mesenteritis. We, however, acknowledge that IgG4-related sclerosing mesenteritis is a separate entity and needs more studies regarding treatment outcomes before evidence-based guidelines can be created. The therapy of choice for IgG4-related disease is corticosteroid treatment, and it was effective in some of the published cases [7, 8]; however, it should be noticed that most of the cases reported in the literature [4, 6-8, 10] underwent laparotomy with complete or partial resection of the lesion and surgery alone was sufficient for local control in these cases with different follow-up time.

## Conclusions

Our case represents a case of IgG4-related sclerosing mesenteritis presenting as a mesenteric mass, which meets the histologic criteria for this rare disease and is compatible with several other cases described in the literature. More observations are needed to better define the natural course and optimize treatment for this illness.
